# Soil properties and carbon and nitrogen pools in a young hillside longan orchard after the introduction of leguminous plants and residues

**DOI:** 10.7717/peerj.5536

**Published:** 2018-08-30

**Authors:** Huimin Xiang, Yuan Zhang, Hui Wei, Jia-en Zhang, Benliang Zhao

**Affiliations:** 1 Department of Ecology, College of Natural Resources and Environment, South China Agricultural University, Guangzhou, Guangdong, China; 2 Key Laboratory of Agro-Environment in the Tropics, Ministry of Agriculture, South China Agricultural University, Guangzhou, Guangdong, China; 3 Guangdong Engineering Research Center for Modern Eco-agriculture and Circular Agriculture, South China Agricultural University, Guangzhou, Guangdong, China

**Keywords:** Soil properties, Young hillside longan orchards, C pool, Legume, N pool

## Abstract

The intensification of young hillside *Dimocarpus longan* orchard cultivation has led to increase soil erosion and decrease soil fertility in South China. Leguminous crops are often used for improving soil properties. An approximately 2-year-long field experiment in lateritic soil in South China was conducted to evaluate the effects of legume introductions on soil properties and carbon (C) and nitrogen (N) pools. Two leguminous and one non-leguminous plant species, including *Arachis hypogaea* L. (a leguminous oilseed crop species, DA), *Stylosanthes guianensis* (a perennial herbaceous leguminous species, DS) and *Lolium perenne* L. (an annual non-leguminous forage species, DL), were introduced into a *D. longan* orchard as three treatments and compared to the monoculture of *D. longan* (the control, D0). And the harvested biomass residues of the three cover plants were returned to their corresponding plots as green manure. Soil samples were collected from depths of 0–10 and 10–20 cm approximately 2 years after treatment application. The results showed that, compared with D0, DA significantly improved the contents of soil available phosphorus, dissolved organic carbon (DOC), total nitrogen, ammonium and the N pool. In addition, DS significantly increased the contents of DOC, microbial biomass carbon and ammonium in the soil. However, DL did not affect any soil properties or the C and N pools. In addition, neither DA nor DS altered the soil bulk density or the contents of available nitrogen, total organic carbon and the C pool. The improvement of soil properties by DS and DA was positively correlated with the plant residues amount, plant N content but negatively correlated with the plant C:N ratios. Besides, the plant growth of longan was significantly improved by DA. In conclusion, compared with that of *S. guianensis*, the introduction of *A. hypogaea* L. was more helpful for restoring and improving soil properties, N pool and longan growth within the young hillside orchard in South China.

## Introduction

Many hills and slopes are present in South China. The area of existing slope land in Guangdong Province is 13.51 × 10^4^ km^2^, which accounts for 75.94% of the total land area (Zhuo et al., 2004). Mountainous slope land regions are often developed for growing fruits, e.g., longans and lychees (Peng, 2001). However, new orchards, especially those approximately 1–3-years-old, are often full of weeds due to hot and rainy climate during the spring in South China. To avoid influencing the growth of young fruit trees, farmers tend to remove weeds. However, when the weeds are absent, the soil is bare and easily eroded by surface runoff. Due to the high temperatures and rainfall in South China, soil erosion in this area is high. At present, the eroded area of slope land in Guangdong Province is approximately 1.14 × 10^4^ km^2^, which accounts for 79.74% of the total area of soil erosion in this province (Zhuo et al., 2004). Soil erosion results in decreased soil fertility and lower ecosystem productivity in the subtropical area. Therefore, it is important to explore effective methods to reduce the soil erosion of slope land in South China.

The introduction of leguminous species can be an effective and sustainable approach to control soil erosion and improve soil fertility (Peoples, Herridge & Ladha, 1995; Spehn et al., 2002; Hauser, Nolte & Carsky, 2006; Herridge, Peoples & Boddey, 2008; Paynel et al., 2008; Wang et al., 2010). The presence of vegetation provides physical protection of the soil, reduces surface runoff and surface shear stress (Li et al., 2006, 2007; DuPont, Ferris & Van Horn, 2009; Yuan et al., 2016); thus, vegetation prevents soil erosion and reduces nutrient losses (Brandt, 1988; Woo & Luk, 1990; Ulen, 1997; Bechmann et al., 2009). In addition, leguminous crops often can fix dinitrogen (N_2_) from the atmosphere and therefore represent an important option for improving nitrogen (N) supplies to maintain soil fertility (Buerkert, Bationo & Dossa, 2000; Bilgo et al., 2007; Wichern et al., 2008; Zhou et al., 2011). It is reported that intercropped legumes can store nitrogen with the amount between 40 and 100 kg N ha^−1^ in the aboveground part (Amossé et al., 2014; Duchene, Vian & Celette, 2017). And also it can increased soil C sequestration and storage (Drinkwater, Wagoner & Sarrantonio, 1998; Li et al., 2016; Wu et al., 2017).

Crop straw is another accessible and common organic material to help farmers improve soil fertilities. The application of plant residues to soil stimulates soil microbial growth and activity, with the subsequent mineralization of plant nutrients (Eriksen, 2005; Randhawa et al., 2005), and increases soil fertility and quality (Tejada, Hernandez & Garcia, 2009). Crop residues incorporated by tillage not only influence soil biological properties, but also affect soil physical and chemical properties (Wang et al., 2015; Mu et al., 2016). Researches showed that crop residue incorporation into soil increases soil organic matter (SOM), aggregate stability and inorganic-N content (Kitou & Yoshida, 1994; Malhi et al., 2012; Mu et al., 2016), greatly affect SOM and N cycling (Bever, 2003). However, the influence of plant residues on soil properties depends on the amount, return frequency and characteristics of the added plant residues.

To our knowledge, the studies about plant introduced in orchard (also called agroforestry system) on soil properties and fruit production was conducted worldwide (Rey, 2011). And the results showed that plant introduced in orchard could bring positive or negative ecological consequences. Some researchers observed that plant introduced in orchard increasing the soil nutrients contents (Sánchez et al., 2007), stimulating soil biological activities (Hoagland et al., 2008; Ramos et al., 2011), and promoting fruits plants growth and production (Lipecki & Berbec, 1997). In contrast, other studies found that the introduced plant in orchard inevitably absorbed a part of water and nutrients, and result in competitions with fruit trees, at last declined the tree growth and fruit productivity (Hoagland et al., 2008; Gucci et al., 2012). However, in China, clean tillage (i.e., weeding to keep the orchard floor clean) is still a popular practice for orchard floor management (Wang et al., 2015; Wei et al., 2018). And the related research in China is limited. Moreover, there is lack of the studies about the comprehensive effects of plant introduced and residues return on soil properties and fruit plant growth. Knowledge is in particular scarce on different plants introduced and residues return on soil properties and C and N pools in a young hillside orchard.

To determine whether the introduction of leguminous species and their residues return improve soil fertility within young hillside orchards, we introduced two leguminous plant species (*Arachis hypogaea* L. and *Stylosanthes guianensis*) and one non-leguminous species (*Lolium perenne* L.) into a young hillside longan orchard in March 2015 and assessed the effects of these species introduction and residues return on soil nutrients and carbon (C) and N pools in a field experiment. And also we determined the correlations between soil properties and leguminous plant C:N characteristics. The ultimate goal was to identify sustainable management practices for the restoration and improvement of soil properties within young hillside orchards in South China. We hypothesized that intercropping leguminous plants and residues return help improve soil properties and increase C and N pools.

## Materials and Methods

### Field experiments

This study was conducted at the South China Agricultural University research station in Zengcheng City, Guangdong Province, South China (23°14′N, 113°38′E); the elevation of the site is 30 m above sea level. The region is characterized by a typical subtropical humid monsoon climate that presents distinct seasonal patterns. The mean annual precipitation is 1967.8 mm; approximately 80% occurs during the warm wet season (April–September), and 20% occurs during the cool dry season (October–March). The mean annual temperature is 21.7 °C; the maximum and minimum monthly mean temperatures are 28.7 °C in July and 13.3 °C in January, respectively. The accumulated temperature greater than 10 °C throughout the year ranges from 7,000 to 7,911 °C. The mean annual solar radiation is 4482.3 MJ m^−2^, and the annual sunshine duration is 1707.2 h. The soil is lateritic red earth. The soil pH, SOM, total nitrogen (TN), available nitrogen (AN), available phosphorus (AP) and available potassium (AK) in the surface layer (0–10 cm) are 5.51, 18.21 g kg^−1^, 1.29 g kg^−1^, 75.57 mg kg^−1^, 18.32 mg kg^−1^ and 25.22 mg kg^−1^, respectively.

The *Dimocarpus longan* orchard in this experiment was established in 2013. The treatments in our study were conducted in March 2015. Four treatments were established in this study: (1) a nil-crop control (monoculture of *D. longan*, no other plant was introduced, D0); (2) a *D. longan* orchard introduced with a leguminous oilseed peanut (*A. hypogaea* L., DA) crop; (3) a *D. longan* orchard introduced with a perennial herbaceous legume (*S. guianensis*, DS) crop; and (4) a *D. longan* orchard introduced with an annual non-leguminous forage (*L. perenne* L., DL) crop. A completely randomized block design was established; each plot was 144 m^2^ (9 × 16 m) per treatment, and there were three field replications. Each plot consisted of six longan trees that were planted in two rows; each row contained three trees. The distance between the two rows of longan trees was six metres. Inter-plant distance within the same row was five metres. All of the introduced plants species were sown in March 2015. For the DA plot, two seasons of peanut (*A. hypogaea* L.) crops were planted within a single year; they were sown in March and August of each year and were harvested in June and December, respectively. In the DA plots, the distance between different rows of peanut seedling was 25 cm, and the distance within the same seedling row was 20 cm; each ridge contained 10 seedlings. The ridges were spaced 50 cm apart, and there were five ridges per plot. *S. guianensis* and *L. perenne* L. were seeded in rows, which were spaced 50 cm and 25 cm apart, respectively. *S. guianensis* was sown in March 2015. Although *S. guianensis* was a perennial herbaceous, we harvested it once in December 2015. *L. perenne* L. was sown in March 2015 and harvested in December 2015 and May 2016, and then it was second sown in August 2016.

**Figure 1:**
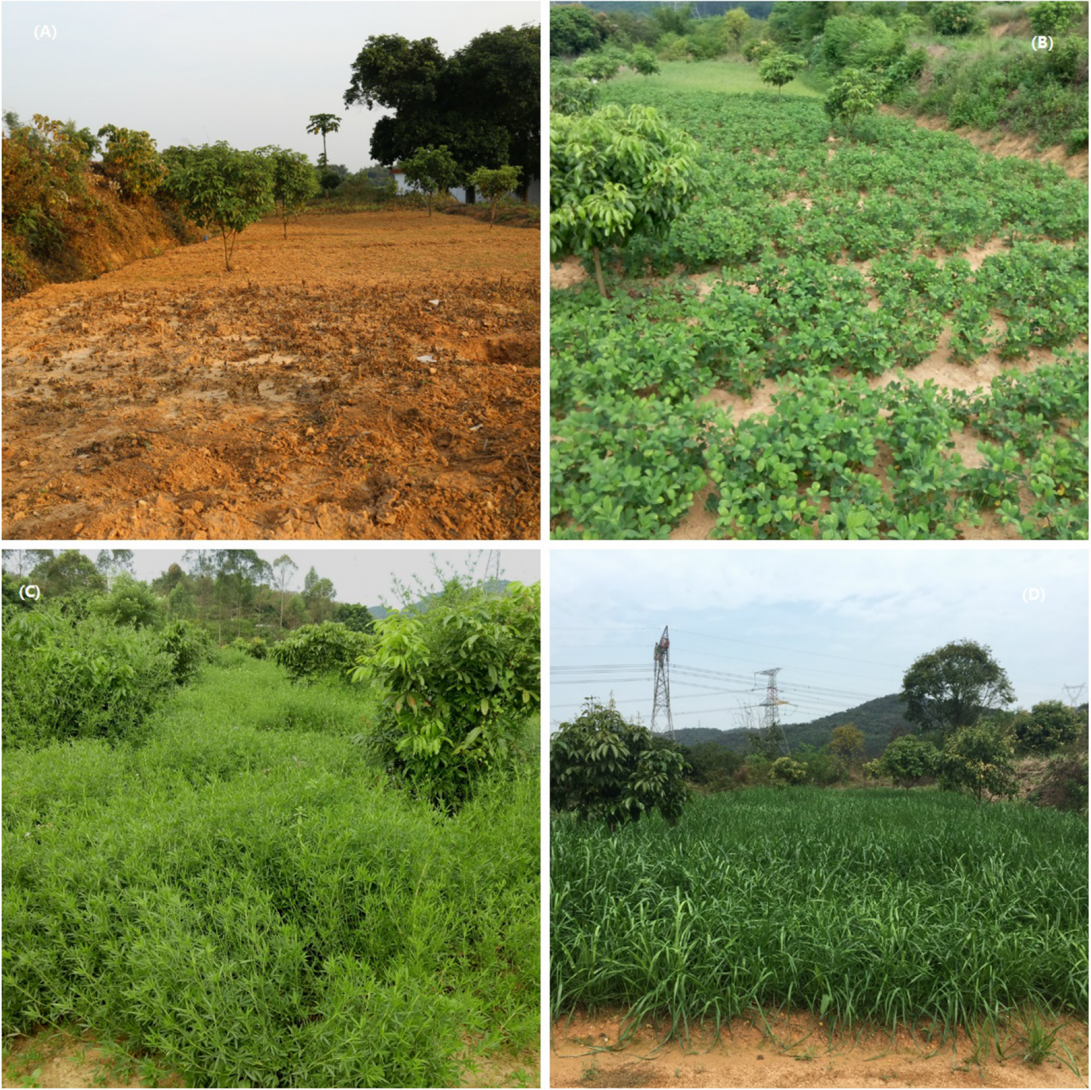
Four treatments within the field experiment. (A) is the control; (B–D) show the introductions of *Arachis Hypogaea* L. (a leguminous oilseed crop species (peanut)), *Stylosanthes guianensis* (a perennial herbaceous leguminous crop species) and *Lolium perenne* L. (an annual non-leguminous forage species) into young hillside longan orchards, respectively. Photos by Yuan Zhang.

Before the above three plant species were seeded, 600 kg ha^−1^ compound fertilizer (% N-% P_2_O_5_-% K_2_O = 15-15-15) was applied as a basal fertilizer to all of the treatments. Thereafter, no other chemical fertilizers were applied to these treatments. When *A. hypogaea* L., *S. guianensis* and *L. perenne* L. were harvested, all of their straw was subsequently used as green manure. The straw was cut and mulched on the corresponding plots; the mulch was then incorporated into the surface soil when the land was plowed. Images of this field experiment are shown in Fig. 1.

### Soil and plant sampling

Soil samples were collected in December 2016. Each sample consisted of mixture of five individual soil cores that were collected at depths of 0–10 and 10–20 cm. The soil samples were sieved through a two mm mesh to thoroughly homogenize and remove any visible roots, plant residue and stones. A portion of each soil sample was stored at 4 °C for measuring dissolved organic carbon (DOC) and microbial biomass carbon (MBC) contents, and the remaining soil was dried at room temperature for measuring soil physicochemical properties.

Plant total aboveground dry weight (TDW) of the introduced plant species was measured from an area of 1 × 1 m; three replications were randomly sampled per plot. All vegetation within each treatment was cut and transported to the laboratory. The samples (including both leaves and stems) were oven-dried at 85 °C to constant weight and then measured, after which they were ground for measuring the C and N contents.

Before the soil samples were collected, plant residues of DA, DS and DL were applied three, one, and two times, respectively. And the total TDW (TTDW) that returned into soil during this study was shown in Table 1.

**Table 1:** TDW, C and N contents, and the C:N ratio in the three introduced plant species.

Type	TDW (t ha^−1^)	TTDW (t ha^−1^)	W (C) (%)	W (N) (%)	Total N-input (t ha^−1^)	C:N ratio
*Arachis hypogaea* L.	4.78 (0.29)^c^	14.34 (0.88)^a,b^	40.84 (0.34)^b^	3.99 (0.13)^a^	57.40 (5.41)^a^	10.26 (0.27)^c^
*Stylosanthes guianensis*	11.60 (1.21)^a^	11.60 (1.21)^b^	40.81 (0.13)^b^	3.45 (0.11)^b^	39.82 (3.09)^b^	11.84 (0.39)^b^
*Lolium perenne* L.	8.37 (0.61)^b^	16.75 (1.21)^a^	42.20 (0.44)^a^	2.25 (0.09)^c^	37.60 (2.28)^b^	18.79 (0.64)^a^

**Notes:**

TDW indicate total aboveground dry weight, TTDW indicate total TDW during the whole experiment.

Different letters indicate significant differences between treatments, and numbers in brackets represent the standard error of the means (*n* = 3) at *p* < 0.05.

### Laboratory analyses

The soil bulk density (BD) was measured gravimetrically. The soil total organic carbon (TOC) was measured using dichromate (0.8M) heating oxidation (Lu, 1999).

The MBC concentrations were determined using the CH_3_Cl fumigation–K_2_SO_4_ extraction method (Wu et al., 1990). Briefly, two portions of each fresh soil sample were weighed in containers. One container was fumigated with ethanol-free CH_3_Cl for 24 h, while the other container was not fumigated and served as the control. After fumigation, K_2_SO_4_ solution was used to retain the extracted C, after which the supernatants were analyzed to determine the C content. The MBC content was calculated using an extraction coefficient of 0.45 (Brookes et al., 1985; Wu et al., 1990). The DOC was extracted by deionized water using a soil: water ratio of 1:2, and the final DOC content was determined using dichromate (0.8 mol L^−1^) heating oxidation (Lu, 1999).

The soil TN, ammonium and nitrate contents were assayed as described by Bao (2000). The soil TN content was determined using the Kjeldahl method, and for the ammonium and nitrate contents, fresh soil samples were extracted in 2M KCl using a soil:solution ratio of 1:5. The extracts were then analyzed using colorimetry and ultraviolet spectrophotometry to determine the ammonium and nitrate contents, respectively. The soil AN was determined using the alkaline hydrolysis diffusion method (Lu, 1999). The soil AP was extracted by NH_4_F and was measured by Mo-Sb colorimetry (UV-754; UNICO, Shanghai, China) (Bray & Kurtz, 1945). The AK was extracted by NH_4_OAc and then subsequently measured by flame photometry (Jones, 1973).

The plant C content was determined using the Walkley–Black method of K_2_Cr_2_O_7_ oxidization and FeSO_4_ titration. The plant N content was measured using Kjeldahl digestion and flow injection analysis.

### Statistical analysis

The experimental data were evaluated by the analysis of variance (ANOVA). One-way ANOVA together with Duncan’s multiple comparison test was used to compare means between plots treated with D0, DA, DS and DL in each of the two soil layers. Bivariable Pearson correlation was used to detect significant relationships between soil properties and the plant C and N contents. The significance level was set at *p* < 0.05. All statistical tests were performed using SPSS 13.0, and all graphs were generated using SigmaPlot 10.0 (Systat Software Inc., San Jose, CA, USA).

The TN-input was measured using the following equation:

$${\rm{Total}}\;{\rm{N - input}}\;{\rm{ = }}\;{\rm{N}}\;{\rm{concentration}}\;{\rm{ \times }}\;{\rm{TTDW}}$$

Where TTDW that returned into soil during the whole experiment.

The C and N pools were measured using the following equation (Batjes, 1996):

$${{\rm{Y}}_{{\rm{pool}}}}\left({{\rm{Mg h}}{{\rm{a}}^-}^{\rm{1}}} \right){\rm{ = X \times BD}} \times th \times 1{{\rm{0}}^{-{\rm{1}}}}$$

where X is the C or N content (g kg^−1^), BD (Mg m^−3^), and *th* is the thickness of the soil layer (cm).

## Results

### TDW and the C and N contents of the introduced plant species

Significant differences in TDW, TTDW, plant C and N contents, TN-input and the plant C:N ratio were observed among the three introduced plant species (*p* < 0.05, Table 1). The TDW of these plant species followed the order of *S. guianensis* > *L. perenne* L. > *A. hypogaea* L. However, due to the different return frequency of these three plant residues, TTDW was in the order of *L. perenne* L. (16.75 t ha^−1^) > *A. hypogaea* L. (14.34 t ha^−1^) > *S. guianensis* (11.60 t ha^−1^). And the C content in *L. perenne* L. was significantly greater than that in the other two plant species. However, the N content in *A. hypogaea* L. was the greatest; this content was higher than that in both *S. guianensis* and *L. perenne* L. by 15.65% and 77.33%, respectively (*p* < 0.05). Consequently, the TN-input in *A. hypogaea* L. was the largest among the three introduced plants, and it was significantly greater than this in *S. guianensis* and *L. perenne* L. by 44.15% and 52.66%, respectively. The plant C:N ratio followed the order of *A. hypogaea* L. (10.26) < *S. guianensis* (11.84) < *L. perenne* L. (18.79). The C:N ratio in *A. hypogaea* L. was 13.34% and 18.79% lower than that in *S. guianensis* and *L. perenne* L. (*p* < 0.05). In addition, the C:N ratio in *S. guianensis* was 36.99% lower than that in *L. perenne* L. (*p* < 0.05).

### Soil C and N fractions and other properties

The results showed that the introduction of plants into the young orchard did not significantly alter the soil BD and AN contents (*p* > 0.05, Table 2) but significantly influenced the soil AP content (*p* < 0.05, Table 2). DA significantly increased the soil AP content in both soil layers. In the surface soil layer (0–10 cm), compared with that in DS, DL and D0 (the control), the soil AP content in DA increased by 75.53%, 116.70% and 111.08%, respectively. In the subsurface soil layer (10–20 cm), compared with that in DS, DL and D0, the AP content in DA increased by 122.59%, 125.37% and 151.44%, respectively. Compared with D0, DA did not significantly reduce the soil AK content; however, DS and DL significantly reduced it.

**Table 2:** Soil physiochemical properties in the surface (0–10 cm) and subsurface (10–20 cm) soil layers in the control and plant introduction plots.

Soil layer	Treatment	BD^a^ (g cm^−3^)	AN^b^ (mg kg^−1^)	AP^b^ (mg kg^−1^)	AK^b^ (mg kg^−1^)
0–10 cm	D0^c^	1.56 (0.06)^a^	42.99 (0.90)^a^	15.80 (1.75)^b^^,^^d^	28.00 (3.32)^a^
	DA^c^	1.52 (0.03)^a^	54.00 (2.00)^a^	33.35 (3.09)^a^	31.93 (2.92)^a^
	DS^c^	1.51 (0.04)^a^	54.80 (3.98)^a^	19.00 (0.99)^b^	18.05 (2.26)^b^
	DL^c^	1.56 (0.03)^a^	49.77 (3.44)^a^	15.39 (2.16)^b^	18.50 (1.88)^b^
10–20 cm	D0	1.62 (0.02)^a^	39.17 (1.59)^a^	12.19 (0.24)^b^	20.34 (3.89)^a^
	DA	1.53 (0.05)^a^	48.37 (5.36)^a^	30.65 (4.80)^a^	14.26 (1.64)^a^^,^^b^
	DS	1.50 (0.05)^a^	40.62 (4.14)^a^	13.77 (1.74)^b^	9.87 (0.53)^b^
	DL	1.50 (0.04)^a^	43.86 (2.81)^a^	13.60 (2.03)^b^	12.25 (1.01)^b^

**Notes:**

a BD indicate bulk density.

b AN, AP and AK indicate soil available nitrogen, available phosphorus and available potassium, respectively.

c D0 denotes the control monoculture of *D. longan*. DA, DS and DL indicate that *Arachis hypogaea* L., *Stylosanthes guianensis* and *Lolium perenne* L. were introduced into longan orchards, respectively.

d Different letters within the same column indicate significant differences between treatments within the same soil depth, as tested by one-way analysis of variance (ANOVA) (*p* < 0.05). Numbers in parentheses represent the standard error of the means (*n* = 3).

**Figure 2:**
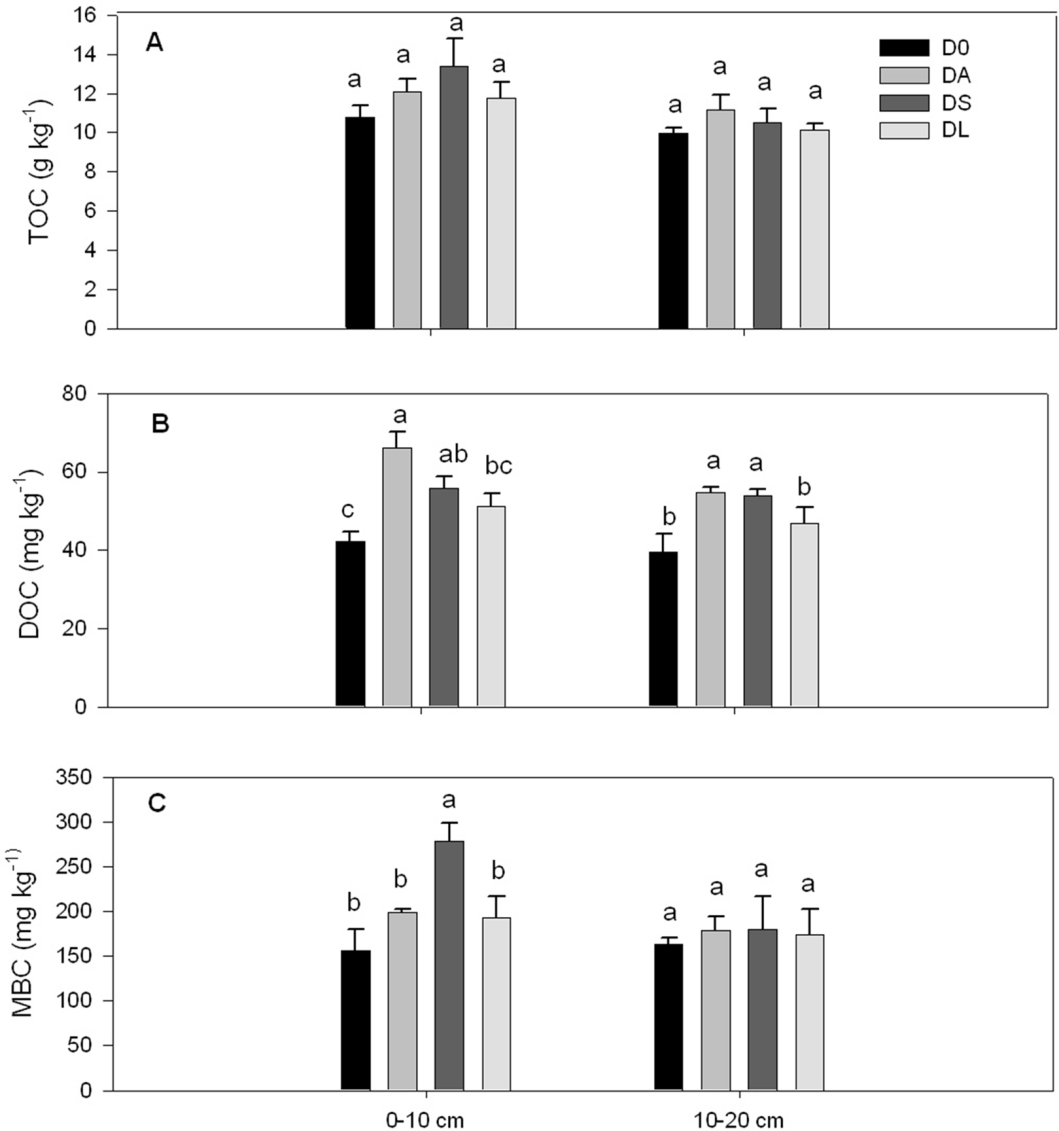
Contents of total organic carbon (TOC; A), dissolved organic carbon (DOC; B) and microbial biomass carbon (MBC; C) in the surface (0–10 cm) and subsurface (10–20 cm) soil layers of the control and plant introduction plots. D0 denotes the control monoculture of *D. longan*. DA, DS and DL indicate that *Arachis hypogaea* L., *Stylosanthes guianensis* and *Lolium perenne* L. were introduced into longan orchards, respectively. The bars indicate the means, and the error bars indicate the standard errors (*n* = 3). Different lowercase letters indicate significant differences at the *p* < 0.05 level between treatments within each soil layer.

The introduction of the plants into the young orchard significantly affected the soil DOC (0–10 and 10–20 cm) and MBC contents (0–10 cm) (*p* < 0.05, Figs. 2B and 2C) but did not affect the TOC content (*p* > 0.05, Fig. 2A). Compared with D0, both DA and DS significantly promoted the DOC content in the surface and subsurface soil layers (0–10 and 10–20 cm), but no significant differences were observed between DA and DS. Moreover, DS significantly increased the MBC content in the surface soil layer (*p* = 0.014, Fig. 2C) but did not significantly alter the MBC content in the subsurface soil layer (*p* = 0.967, Fig. 2C).

**Figure 3:**
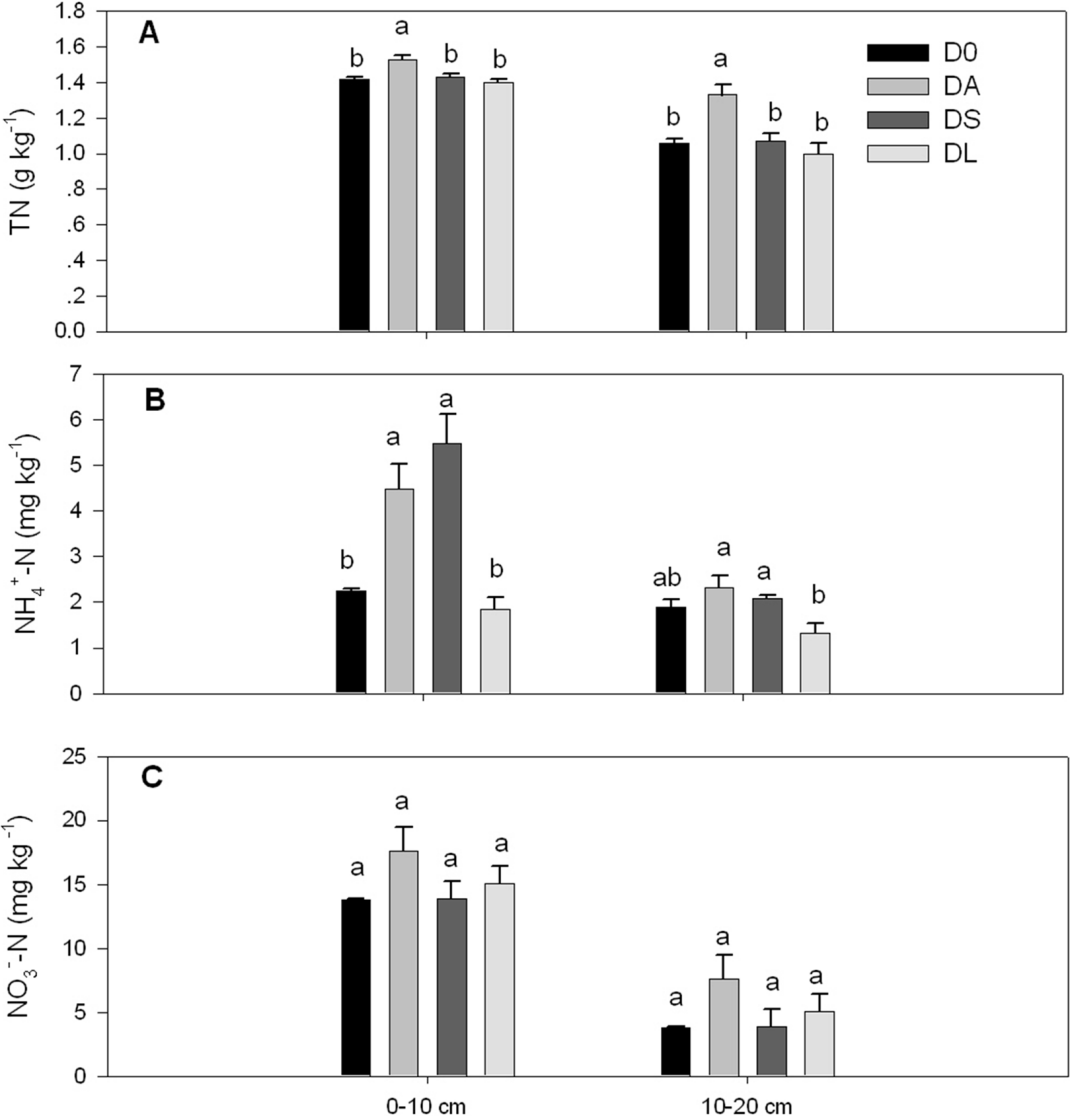
Contents of total nitrogen (TN; A) and N fractions, including ammonium (B) and nitrate (C), in the surface (0–10 cm) and subsurface (10–20 cm) soil layers of the control and plant introduction plots. D0 denotes the control monoculture of *D. longan*. DA, DS and DL indicate that *Arachis hypogaea* L., *Stylosanthes guianensis* and *Lolium perenne* L. were introduced into longan orchards, respectively. The bars indicate the means, and the error bars indicate the standard errors (*n* = 3). Different lowercase letters indicate significant differences at the *p* < 0.05 level between treatments within each soil layer.

Among the four treatments, DA significantly increased the TN content in both the surface and subsurface soil layers (0–10 and 10–20 cm) (*p* < 0.05, Fig. 3A). In addition, DA and DS significantly improved the ammonium content in the surface soil layer (0–10 cm) (*p* < 0.05, Fig. 3B). In particular, the ammonium content in DA and DS increased by 98.67% and 142.48% compared with D0. However, no significantly differences were observed for nitrate contents (*p* > 0.05, Fig. 3C).

### Soil C and N pools

**Figure 4:**
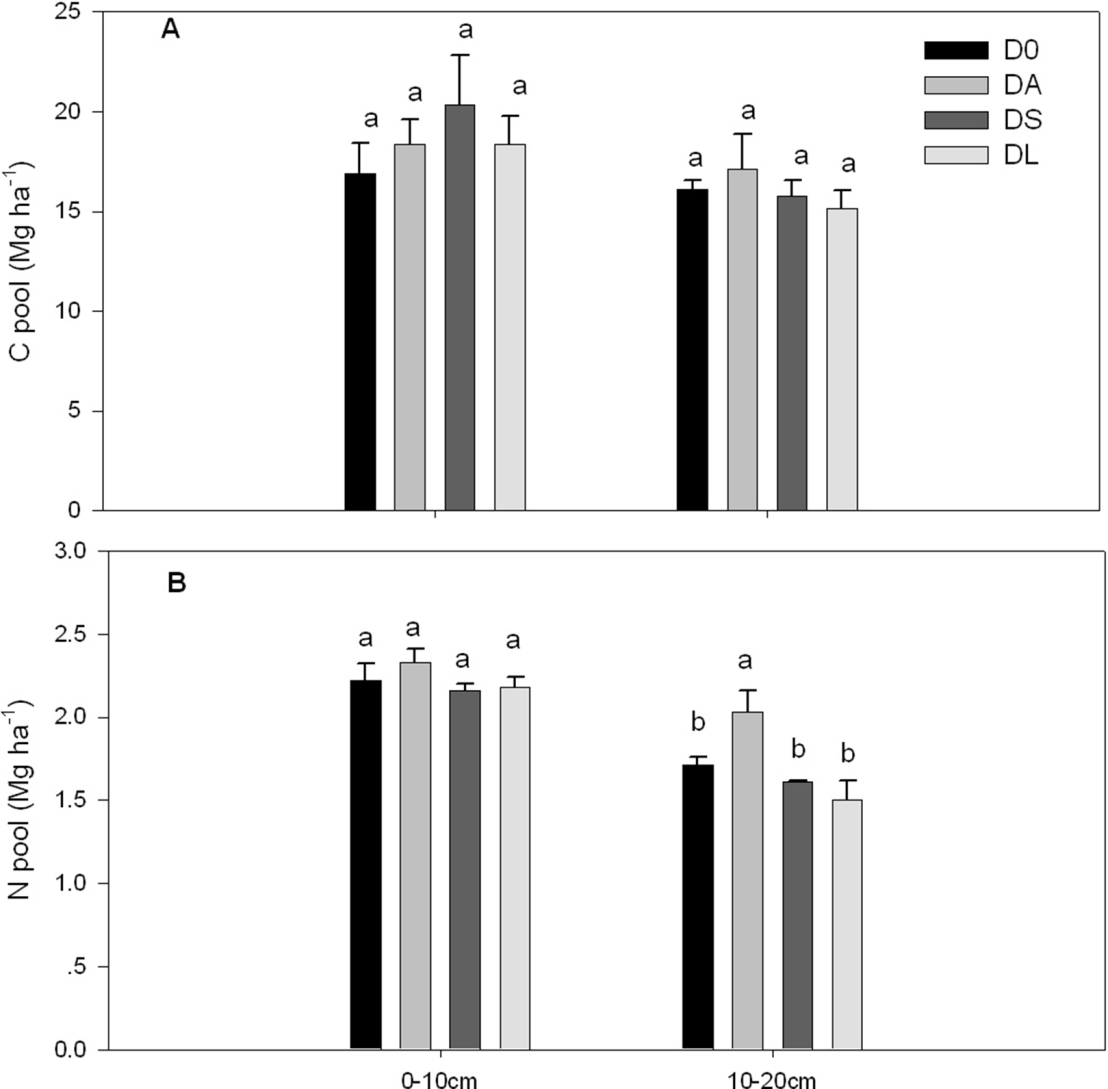
C and N pools in the surface (0–10 cm) and subsurface (10–20 cm) soil layers of the control and plant introduction plots. D0 denotes the control monoculture of *D. longan*. DA, DS and DL indicate that *Arachis hypogaea* L., *Stylosanthes guianensis* and *Lolium perenne* L. were introduced into longan orchards, respectively. The bars indicate the means, and the error bars indicate the standard errors (*n* = 3). Different lowercase letters indicate significant differences at the *p* < 0.05 level between treatments within each soil layer.

The results showed that the introduction of plants into the young hillside orchards significantly affected the soil N pool in the subsurface soil layer (10–20 cm) (*p* < 0.05, Fig. 4B) but did not affect the soil C pool in the surface and subsurface soil layer (*p* > 0.05, Fig. 4A). Compared with that in D0 (control), DS and DL, the N pool in DA significantly increased by 18.71%, 26.09% and 35.33%, respectively, in the subsurface soil layer (10–20 cm) (*p* = 0.017, Fig. 4B). However, no significant differences were observed between D0, DS and DL.

### Correlations between plant C and N contents and soil properties

The soil TOC, MBC, nitrate and AN contents were not significantly correlated with tested plant variables such as plant TDW, C and N contents, and the C:N ratio (*p* > 0.05, Table 3). However, the soil DOC, TN, NH_4_^+^-N and AP contents were significantly positively correlated with plant N content, but negatively correlated with the plant C:N ratio. In addition, the soil AK content was significantly negatively correlated only with plant TDW (*p* < 0.05, Table 3).

**Table 3:** Correlations between soil properties and the plant C:N variables.

	Plant TDW	Plant C	Plant N	Plant C:N ratio
TOC	0.280	−0.100	0.113	−0.137
DOC	−0.635	−0.353	0.815**	−0.745*
MBC	0.650	−0.532	0.165	−0.278
TN	−0.527	−0.473	0.752*	−0.666
NH_4_^+^-N	0.097	−0.615	0.821**	−0.857**
NO_3_^−^-N	−0.568	0.040	0.360	−0.278
AN	0.004	−0.357	0.355	−0.345
AP	−0.598	−0.372	0.760*	−0.678*
AK	−0.785*	−0.271	0.661	−0.545

**Notes:**

Numbers in the cells are correlation coefficients.

TDW, total aboveground dry weight; TOC, total organic carbon; DOC, dissolved organic carbon; MBC, microbial biomass carbon; TN, total nitrogen; AN, available nitrogen; AP, available phosphorus; AK, available potassium.

^*^p < 0.05.

^**^p < 0.01.

### Plant growth of longan after legume and non-legume plants introduced

Significant differences in the plant growth of longan were found after the introduction of legume and non-legume species (*p* < 0.05, Table 4). The results showed that the introduction of legume-plant *A. hypogaea* L. into the young hillside orchards significantly increased the growth of longan plant (*p* < 0.05). The diameter at breast height of longan plant in DA was significantly higher in the percentage of 18.83%, 53.16% and 67.11% than this in DS, DL and D0, respectively. The crown size of DA was also increased by 7.14%, 25.70% and 26.4% compared with DS, DL and D0, respectively. However, there was no significant difference between DA and DS, DS and D0 and DL and D0.

**Table 4:** Plant growth of longan in the control and plant introduction plots.

Treatment	Diameter at breast height (mm)	Height (m)	Crown size (m)
D0	50.86 (3.87)^b^	1.77 (0.10)^a^	1.78 (0.14)^b^
DA	84.99 (13.75)^a^	1.96 (0.19)^a^	2.25 (0.15)^a^
DS	71.52 (4.81)^a,b^	1.91 (0.07)^a^	2.10 (0.09)^a,b^
DL	55.49 (3.85)^b^	1.85 (0.10)^a^	1.79 (0.08)^b^

**Note:**

D0 denotes monoculture of *D. longan* (control). DA, DS and DL indicate that *Arachis hypogaea* L., *Stylosanthes guianensis* and *Lolium perenne* L. were introduced into longan orchards, respectively. Different lowercase letters indicate significant differences at the *p* < 0.05 level between treatments.

## Discussion

Soil TOC content, C fractions (i.e., DOC and MBC) and C pool were analyzed to detect changes in soil C, as soil organic carbon (SOC) is responsible for soil function and the sustainability of agricultural ecosystems (Chen & Xu, 2008; Xu et al., 2009). However, in this study, no significant differences in TOC contents were found between treatments. Different from TOC, soil labile organic C is the most active fraction of SOC and acts as an indicator of potential microbial activity, which is sensitive to land use and management (Needelman et al., 1999; Schloter, Dilly & Munch, 2003; Rovira, Jorba & Romanyà, 2010). In the present study, our results showed that both of the two leguminous plant species introduced into the young orchard significantly increased soil DOC contents in the surface and subsurface soil layers but that non-leguminous plants species did not alter DOC contents. In addition, among the four treatments, *S. guianensis* introduction improved the soil MBC content in the surface soil layer. The increase in labile C fractions in DA and DS can be attributed to two factors. First, the characteristics of leguminous crops benefit improvement of soil labile organic C contents. As we know, active fractions of SOC are closely associated with crop residue biomass and crop species characteristics (Coppens, Merckx & Recous, 2006; Frazão et al., 2010). In the present study, although the TTDW of DA and DS was lower than DL, DA and DS had a higher plant N content and TN-input but lower plant ratio of C:N. This higher quality of crop residues were easily and quickly degraded by soil microbes so that improved the C input nutrients for microorganisms **(**Tian & Shi, 2014**)**. Thus DA and DS added the availability of SOC. Second, because C and N nutrient cycles are closely connected, the increase in N nutrients from DA and DS might prompt improvements in soil active C fractions (Fig. 3).

In addition, soil N fractions (i.e., ammonium and nitrate), TN and the N pool could detect potential changes in the soil TN and availability of nitrogen contents. Consistent with both our hypothesis and the results of previous studies (Bilgo et al., 2007; Wichern et al., 2008; Zhou et al., 2011; Amossé et al., 2014; Duchene, Vian & Celette, 2017), the introduction of leguminous plants and residues increased soil N fractions. In this study, both leguminous plant species (*A. hypogaea* L. and *S. guianensis*) increased the soil ammonium content, but DL did not affect the soil TN and availability of nitrogen contents. This result suggests that the introduction of *A. hypogaea* L. and *S. guianensis* promoted the availability of soil nitrogen in the young orchard, as ammonium and nitrate are generally preferred by plants (Sardans & Peñuelas, 2012). However, only the introduction of *A. hypogaea* L. increased the soil TN content and N pool. This finding suggests that *A. hypogaea* L. can enrich soil TN contents to levels greater than those attainable by *S. guianensis*. The greater TN contents and larger N pools under DA than DS may be due to two reasons. First, *A. hypogaea* L. probably has a greater N-fixation ability than does *S. guianensis*; thus, compared with the latter, the former can accumulate and transfer more N to the soil by fixing atmospheric N in the roots and shoots (Kuo, Sainju & Jellum, 1997; Sainju et al., 2003). Second, as the TTDW that returned into soil during the whole experiment and TN-input of DA was greater than that in DS (Table 1). This results in the higher soil TN and N pools in DA.

In addition, our results indicated that the introduction of herbaceous leguminous (*S. guianensis*) and non-leguminous forage (*L. perenne* L.) species into the young hillside orchards did not improve soil BD and AN but instead reduced the soil AK content. The AK content in DS and DL might be used to improve the aboveground biomass of *S. guianensis* and *L. perenne* L. This phenomenon could have occurred because *S. guianensis* and *L. perenne* L. presented higher plant TDW than did *A. hypogaea* L. (Table 1), and the TDW was negatively correlated with the soil AK content (Table 3). In addition, opposite of our expectations, the soil BD as well as AN content investigated in this study were also not significantly altered by DA in the two soil layers. Therefore, our hypothesis is not supported by our observations in that soil BD and AN content did not increase as a result of the introduction of leguminous crop species. However, surprisingly, the introduction of *A. hypogaea* L. did not reduce the AP content in the soil but instead increased it. These results were opposite with previously ones, as studies have shown that leguminous plants capable of supporting biological nitrogen fixation (BNF) generally have higher phosphorus (P) demands than do graminaceous plants supplemented with mineral N fertilizer (Robson, O’hara & Abbott, 1981). However, BNF can enhance P acquisition in cereal/legume intercropping systems (Duchene, Vian & Celette, 2017). The improvement in soil AP content in DA was largely attributed to two causes. First, the root of *A. hypogaea* L. has been found to release organic acids, including myristic acid, stearic acid, palmitic acid, etc. These organic acids mobilized more P by the acidification of the rhizosphere (Liu et al., 2013). Second, the higher TN-input and lower plant C:N ratio in DA could supply more nutrients for microorganism and accelerate soil microbial growth and activity. This increased the organic acids which released by microorganism and also decomposed more soil organic P into an inorganic form, and finally improved soil P nutrition (Li et al., 2007). This result is very important with respect to P utilization in subtropical areas because AP is greatly lacking within acidic soils in the Subtropics (Barber, 1995; Walker & Syers, 1976).

## Conclusion

The DOC and MBC contents, rather than the TOC content, was significantly improved by the introduction of leguminous plants and residues, implying that the easily usable organic C fractions could have increased as a result of leguminous plants and residues introduction. The introduction of leguminous *A. hypogaea* L. plant and residue significantly increased soil TN contents in the surface and subsurface soil layers. The soil ammonium content, rather than the AN or nitrate content, was significantly greater in the legume introduction plots than in the non-leguminous introduction plots and control plots. Our results suggest that the introduction of legumes into the young hillside orchard improved soil quality and fertility, as the treatments increased soil availability of nitrogen contents and labile C fractions. Moreover, the introduction of *A. hypogaea* L. increased the soil AP content. This result indicated that the introduction of *A. hypogaea* L. plants and residues into the young hillside orchard could not only improve soil N nutrition but also aid in solving the problem of P limitation in the Subtropics. Moreover, the introduction of *A. hypogaea* L. increased the soil N pool in the subsurface soil layer. Most importantly, the growth of longan was significantly improved by the introduction of *A. hypogaea* L. plant and residues return. Therefore, compared with that of *S. guianensis*, the introduction of *A. hypogaea* L. constitutes a better sustainable management practice for the restoration and improvement of soil properties in young hillside orchards in South China.

## Supplemental Information

10.7717/peerj.5536/supp-1 Supplemental Information 1 Raw data of figures and tables.

## Additional Information and Declarations

### Data Availability

The following information was supplied regarding data availability: The raw data are provided in the Supplemental File.
